# Influence of Silicon Carbide on Direct Powder Bed Selective Laser Process (Sintering/Melting) of Alumina

**DOI:** 10.3390/ma15020637

**Published:** 2022-01-15

**Authors:** Asif Ur Rehman, Muhammad Ahsan Saleem, Tingting Liu, Kai Zhang, Fatih Pitir, Metin Uymaz Salamci

**Affiliations:** 1 ERMAKSAN, Bursa 16065, Turkey; Fatih.pitir@ermaksan.com.tr; 2 Department of Mechanical Engineering, Gazi University, Ankara 06570, Turkey; msalamci@gazi.edu.tr; 3 Additive Manufacturing Technologies Research and Application Center-EKTAM, Gazi University, Ankara 06560, Turkey; 4 School of Mechanical Engineering, Nanjing University of Science and Technology, Nanjing 210094, China; ahsansaleem@njust.edu.cn (M.A.S.); Zhangkai@njust.edu.cn (K.Z.); 5 Manufacturing Technologies Center of Excellence-URTEMM A.S., Ankara 06560, Turkey

**Keywords:** additive manufacturing, 3D printing, selective laser sintering/melting (PBSLP), ceramic, composites

## Abstract

The powder bed selective laser process (sintering/melting) has revolutionised many industries, including aerospace and biomedicine. However, PBSLP of ceramic remains a formidable challenge. Here, we present a unique slurry-based approach for fabricating high-strength ceramic components instead of traditional PBSLP. A special PBSLP platform capable of 1000 °C pre-heating was designed for this purpose. In this paper, PBSLP of Al_2_O_3_ was accomplished at different SiC loads up to 20 wt%. Several specimens on different laser powers (120 W to 225 W) were printed. When the SiC content was 10 wt% or more, the chemical interaction made it difficult to process. Severe melt pool disturbances led to poor sintering and melting. The structural analysis revealed that the micro-structure was significantly affected by the weight fraction of SiC. Interestingly, when the content was less than 2 wt%, it showed significant improvement in the microstructure during PBSLP and no effects of LPS or chemical interaction. Particularly, a crack pinning effect could be clearly seen at 0.5 wt%.

## 1. Introduction

Additive manufacturing (AM) has revolutionised many industries, and more are diving in, partially or completely, to accomplish design freedom with reduced time to market [1,2,3,4,5,6,7,8,9]. LPBF is AM method for producing parts and freeform articles in such a way that the manufacturing layer is fused selectively by a high-energy laser beam [10] after powder deposition [11,12,13,14]. LPBF is able to achieve 100 percent density and excellent mechanical properties [15,16,17,18,19,20] for alloys with melting of powder particles. It is of great interest to accomplish direct LPBF for non-weldable materials including ceramics [21,22] due to its utility in many applications. Indirect LPBF of ceramics is achievable using metal, polymer or glass as binder to consolidate parts. These binders can also be removed by debinding in the case of polymers [23]. However, due to the low densities and weak binding strength, the horizon of application is very limited. Direct LPBF can give 100 percent density of ceramics; however, melting–solidification dynamics increase thermal stresses, which make it impossible to obtain consistent ceramic parts. By controlling the laser melt pool and reducing thermal stresses, ceramics parts production could be possible, but clear information on melt pool physics as well as laser–material interaction is needed.

LPBF has the extraordinary cycling rate of heating and cooling which triggers nonequilibrium conditions; new microstructures and material phases are usually formed during the process [24]. It is well established that the LPBF of alloys and metals works on same principle of welding during melting and solidification. It has been extensively studied for fabricating high-performance parts for a variety of metals, including tungsten [25], titanium [26,27,28,29,30], aluminium [31,32,33,34,35], stainless steel [36,37,38] and high-speed steel [39,40]. Welding concepts such as nucleation theory and undercooling [39] have also been used to achieve high-strength metal parts [41,42,43]. Our findings suggest that laser-induced melt pool is also effective.

Controlling the laser-driven melt pool is the key to diversifying the materials processability of LPBF [44,45,46]. The laser beam has a specific direction and intensity along with the mechanical impact it carries due to the energy packets within the beam [47]. Great efforts have been made by researchers to see the laser-induced flow through many techniques, which might be due to one or several of combined effects of matter–light interaction, fluid physics, laser-induced thermodynamics and laser-induced ultrasound. A laser can move liquid with less surface tension very easily. Laser irradiation is not simply heat, typically correlated in LPBF. Lasers may induce several physical phenomena that have been extensively proven in the literature, e.g., momentum transfer is also achievable through indirect transfer by fluorescent photons or direct transfer from incident photons [48], and the momentum of the laser may drive a microfluidic device in high-energy lasers [49,50]. Liquid deformation has been demonstrated through light scattering using a liquid–liquid interface by minimising surface tension at the two-phase boundary using a mixture of two liquids [51]. Matter–light manipulation has also pushed the development of many techniques. Laser beams can transport matter in radiometric force or photopheresis using gas media for low-density materials [52]. Laser irradiation may also induce special flow due to the ultrasound induced by lasers. Fluid motion is controlled by lasers in optical tweezing [53,54]. Fluid mixing [55,56] or generation of droplets [57] can be accomplished, and the chromocapillary effect [58,59] or optothermocapillary effect [60,61,62] can realize the transportation of droplets. Droplet manipulation can also be achieved using these techniques [58,59,60,61,62]. The well-known Marangoni effect is the driving force behind all the chromocapillary or optothermocapillary effects in all these studies, which is quite remarkable.

Additive manufacturing (AM) of ceramics is one of the most widely studied subjects because of its useability in many industrial sectors. Researchers have tried indirect ways to manufacture ceramics with binders (i.e., polymers) that can be removed by the debinding process, but the process may drastically decrease the density of ceramics and also influence mechanical properties [21,23,63,64]. Powder bed selective laser process (sintering/melting) of pure ceramics without any binder or direct PBSLP remains a formidable challenge [21,63,64,65,66,67]. However, direct PBSLP is highly desirable, and researchers have investigated many individual factors which effect the PBSLP process, from pre-heating, surrounding temperature, scanning speed, hatch spacing, laser power, interval time, laser scanning strategy (diagonal, island, zigzag and others), the influence of the non-steady-state melting regime in the scanning track, influence of porosity on fatigue crack initiation, laser pre-heating of ceramic powder, powder particle size, mixed nano and micron-sized powder particles, to various materials [27,63,65,67,68,69,70,71,72]. At this stage, direct PBSLP of ceramic is not mature enough until all the factors affecting it are combined to secure the most efficient quality of ceramic articles [12,14].

Manufacturing of Al_2_O_3_ through PBSLP is challenging and potentially rewarding for its low cost and use in advanced applications. It has also become the focus of recent research because of its remarkable properties such as high hardness, low electrical conductivity, excellent chemical stability, oxidation resistance and high wear resistance [73,74,75,76,77]. Despite these excellent qualities, Al_2_O_3_ ceramics have low flexural strength and fracture. Remarkable improvements have been observed when a small amount of SiC is added to Al_2_O_3_ [78]. The factors responsible for the improvement in mechanical properties of Al_2_O_3_/SiC composites are still under investigation; however, some researchers pointed out that this change is caused by residual stresses created upon cooling around SiC due to the difference in thermal expansion coefficients [78,79,80].

While manufacturing fully dense ceramics directly through PBSLP can generate cracks and other manufacturing defects [81], the material addition can improve the manufacturability [13]. Since SiC has slightly higher sublimation/melting temperature (2700–~3000 °C) than Al_2_O_3_ that is ~2300 °C, it can improve the manufacturability by crack deflection and pinning effect [82].

Alejandro et al. investigated the manufacturing possibility that silicon carbide (SiC) may be processed using direct Powder Bed Selective Laser Processing (PBSLP) and detailed how the laser power and scanning speed must be adjusted such that the scanning temperature was between the sintering and decomposition limitations [83].

Zhang et al. studied at how the surface morphology and melting state of pure Al_2_O_3_ ceramics changed as a result of changes in laser parameters. The laser power was adjusted from 100 to 200 W, while the scan speed was modified from 60 to 90 mm/s. Thermal capillary convection was noticed by the researchers during the SLM procedure, according to the findings. SLM of Al_2_O_3_ slurry produced streak convection and flowing Bénard cells by varying the temperature gradient. The researchers came to the conclusion that it is possible to fabricate slurry ceramic pieces without the need for binders using SLM [84].

In the present paper, the influence of SiC particles on Al_2_O_3_ in PBSLP is investigated. When SiC is used as an additive, it can prevent cracks, mainly through crack pinning and crack deflection. During traditional as well as additive manufacturing methods, Al_2_O_3_ and SiC have many uses in both ways when Al_2_O_3_ is used as a matrix or as an additive in SiC. When Al_2_O_3_ is used as an additive, it can cause liquid phase sintering of SiC at much lower temperatures. There are some similar chemical effects to liquid phase sintering when the SiC is more than 10 wt% that have also been discussed.

## 2. Materials and Methods

Al_2_O_3_ powder with 99% purity and an average particle size of 0.62 µm was used; the chemical composition is given in Table 1. SiC powder has 0.5–0.7 µm average particle size with 99% purity. The preliminary powder mixture with different concentrations of both powders has been prepared in different ratios: Al_2_O_3_ wt% of 99.5, 98, 95, 90 and 80 with SiC wt% of 0.5, 2, 5, 10 and 20, respectively.

Layer Deposition Method

**Figure 1:**
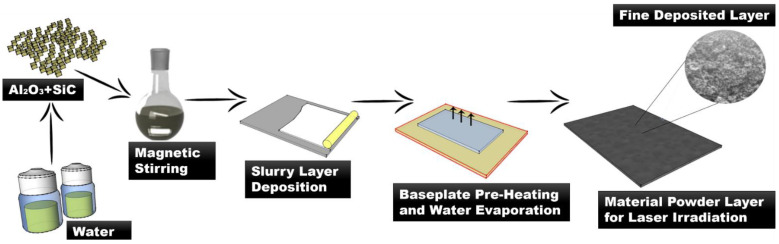
Layer deposition method.

In manufacturing SiC-Al_2_O_3_ composites through PBSLP, ceramic layer deposition of the pure powder is hard to maintain because of the inherent properties of the ceramic powder, so during material layer deposition, we used a two-step deposition methodology as described in Figure 1. Firstly, the powder as per the desired material composition was mixed with water, resulting in a slurry, which was deposited onto the layer movable platforms. The movable system’s platform thickness can be increased from a minimum of 10 μm. The slurry was deposited onto the platform with the layer thickness 50 μm with a powder levelling system equipped with rubber scrapers. Then, the water was evaporated completely by heating the base plate at ~110 °C. The laser irradiation of each layer of the mixed powder directly sinters or melts the ceramic particles. The whole process is repeated until the required number of layers are manufactured as per the requirements of the final article.

Experimental Setup

**Figure 2:**
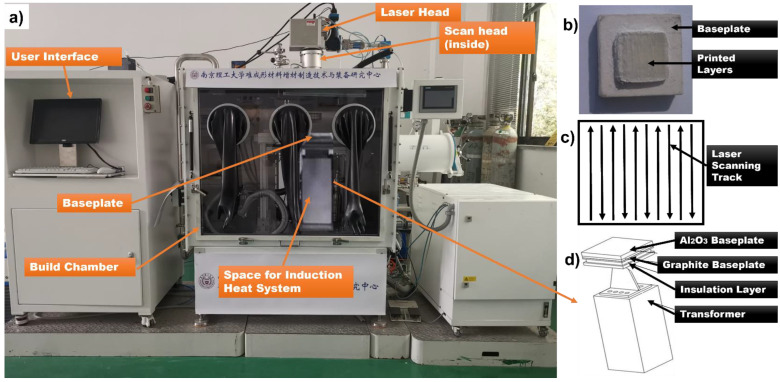
(**a**) Experimental setup. (**b**) Printed layers of ceramic specimen. (**c**) Laser scanning strategy. (**d**) Schematic of induction heating system.

The PBSLP system is equipped with the IPG YLR-500 fibre laser, (IPG Photonics, Oxford, MA, US), which produces a laser beam with the wavelength of 1070 nm and can reach a maximum power of the 500 W in continuous mode. The laser is led through a scanner (SCANLAB intelliSCAN 20, SCANLAB GmbH, Puchheim, Germany). The spot size of the focused laser beam is about 60 μm. The system is also integrated with the induction heating system (20 KW) produced by the Shanghai Bamac, Shanghai, China capable of rapid heating, and the maximum preheating temperature is about 1000 °C. The PBSLP system designed and built for ceramics capable of pre-heating up to 1000 °C has been shown in Figure 2a while the induction heating system mechanism has been broken down in Figure 2d. The printed specimen of Alumina has been shown in Figure 2b while the printing strategy has been shown in Figure 2c.

The experiment was carried out with some variable process parameters, such as laser power (P = 10–50%), while keeping some factors constant, such as the scanning speed (200 mm/min) and the laser hatch distance (50 µm). Parts were printed in the layer after layer pattern, and the layer thickness was kept at 50 µm. The size of every part was kept under the 40 layers. After the manufacturing was completed, the samples were cleaned.

For scanning electron microscopy (SEM) and energy dispersive spectroscopy (EDS) analysis (Oxford Instrument, Bristol, UK), the samples were gold-coated (by the Leica ACE, (Leica Gmbh, Wetzlar, Germany) coater for five minutes). SEM was used to detect and examine the structure of the material.

## 3. Results and Discussions

In the experimental process, two phenomena were involved. Firstly, when the SiC content is in low, there is no chemical interaction, and an improvement can be observed. Secondly, when the SiC content is 10% or above, we found that the increase in SiC content in Al_2_O_3_ can increase the chemical reaction, which leads to high deformations and porosity. It is quite interesting because similar effects can only be found in the literature on liquid phase sintering of SiC. During manufacturing of SiC with the aid of metal oxides, such as Al_2_O_3_, Y_2_O_3_ and other rare-earth oxides, liquid phase sintering of SiC can be achieved at much lower temperatures (1800–1900 °C) [85,86]. The oxide sintering aids react with SiO_2_, which is always present at the surface of SiC particles while forming a silicate melt and enhancing densification. However, oxides interact with SiC with massive gaseous products formation leading to high weight loss and porosity [87]. It is known that Al_2_O_3_ may interact with SiC according to the following reactions [88]:

SiC _(S)_ + Al_2_O_3 (S)_ → Al_2_O _(g)_ + SiO _(g)_ + CO _(g)_ (1)

2SiC _(S)_ + Al_2_O_3 (S)_ → Al_2_O_(S) (g)_ + 2Si _(l)_ + 2CO _(g)_ (2)

3SiC _(S)_ + Al_2_O_3 (S)_ → 2Al _(l)_ + 3Si _(l)_ + 3CO _(g)_ (3)

**Figure 3:**
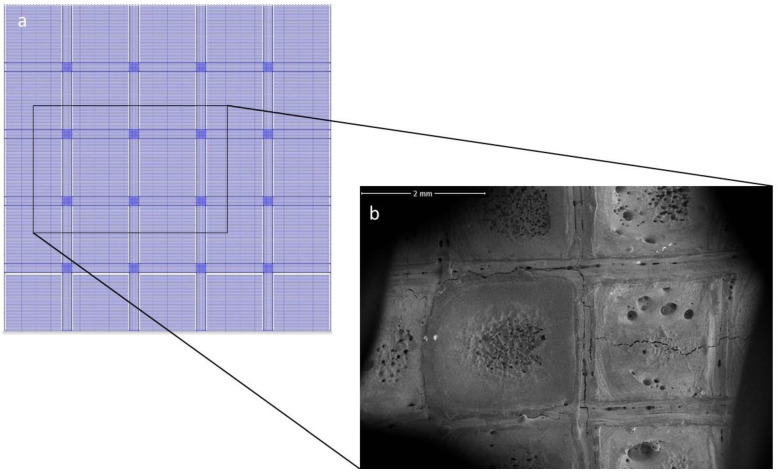
(**a**) Laser scanning strategy. (**b**) SEM of the article in region of the scanning strategy.

In PBSLP, higher content of SiC triggers a chemical reaction to a large quantity which can disturb the melt pool and the surface of the article, as mentioned in Equations (1)–(3). This leads to the unpredictable surface under the same laser parameters for which there is no reaction for the article (under same experimental conditions) with less SiC. The chemical reaction leads to high porosity, increased warping and cracks. The samples of 10% SiC content and beyond were high in the deformation, and the layer size increased due to warping, cracks, deformation and greater porosity, making it challenging to continue to deposit another layer because of the collision between the edges of powder levelling system and the surface of the printed layer of the article. New laser scanning strategies (as shown in Figure 3) were employed to analyse the chemical interaction and its effect of laser-induced melt flow in laser scanning lines, and vice versa. In the laser strategy, small island-like laser scanning lines were used (instead of long scanning lines or so-called zigzag scanning strategy) to decrease the warping and to limit the chemical effects to smaller regions. According to a recent study of the real-time analysis of the melt pool under the laser, the melt pool is in a steady state in the middle of the scanning track and unsteady on the edges of the scanning track [71]. The chemical reaction is affected by the melt pool; it can be seen in Figure 3a,b that when the melt pool is in steady state, the reaction rate is higher as compared to the edges, when the flow is not steady. The reactions mentioned above may also increase with the increase in laser energy density and the SiC content. Apart from the certain conditions mentioned above, other laser parameters also effect this chemical response.

**Figure 4:**
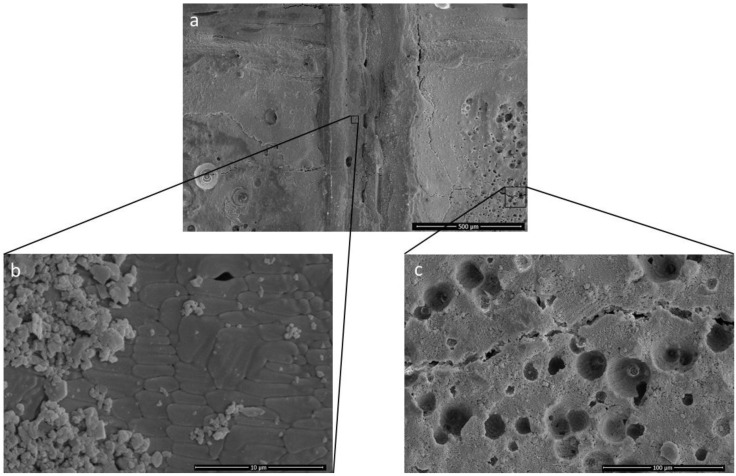
(**a**) Alumina wt% 90, silicon carbide wt% 10 at 140 W laser power. (**b**) Microstructure showing high quality of ceramics where the chemical reaction is low. (**c**) Microstructure showing high porosity due to production of gaseous content where the chemical reaction is high.

When SiC content increases in Al_2_O_3_, the tendency of the chemical reaction increases. As described in Equations (1)–(3), the interaction of Al_2_O_3_ and SiC will ultimately generate the high porosity within the article manufactured by PBSLP. In Figure 4c, the high porosity was seen in the middle of the track (when the laser power was increased to 140 W with the composition of the 90 wt% Al_2_O_3_ and 10 wt% of SiC). However, near the edges of the track of the same article, it can be seen that the additive manufacturing and reaction conditions are entirely different because they show high quality and multi-directional grains of the ceramic article.

**Figure 5:**
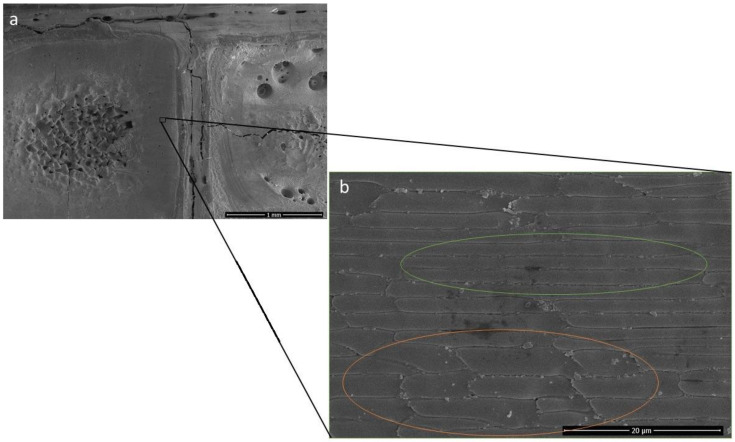
(**a**) Al_2_O_3_ wt% 90, SiC wt% 10 at 160 W laser power. (**b**) Inhibitory effect of grains under laser.

The laser scanning line has a clear impact on the material powder layer due to the momentum of the high energy density of the laser beam. However, the laser path also has a significant effect on the melt pool, and it is responsible for the melt pool flow, but the chemical reaction changes can also hinder the flow. The employed laser scanning strategy makes it easier to see the effects of the laser path and chemical reaction with the state of the melt pool in the SEM, as shown in Figure 3a with small island-like horizontal scanning lines and long continuous vertical and horizontal lines in between. From the analysis of Figure 4a–c as well as Figure 5a,b, it can be deduced that the melt pool state is quite important to control the chemical reaction. In the centre, the laser scanning island (island is a term for small portions of unidirectional laser scanning lines), the chemical change is high compared to the edges of the island, which could be due to the disturbance led by the smaller to higher chemical reaction in the melt pool from the edges. Both the melt pool steady state as well as the chemical interaction are interdependent. When the flow is steady, the chemical interaction starts, and the melt pool starts to become unstable, but when the flow is unsteady, the chemical reaction is less evident.

When the laser melts the material powder during laser scanning, the chemical reaction between the SiC and Al_2_O_3_ inhibits and hinders the laser scan line of the melt pool, and production of SiO particle can also be seen at the surface as a result of the reaction. The silicon oxide particle is more abundantly available on the surface where the melt pool is hindered (orange highlighted area in Figure 5b) and less available where the melt pool is continuous (green highlighted area in Figure 5b), which provides a strong argument for the possible chemical reaction.

**Figure 6:**
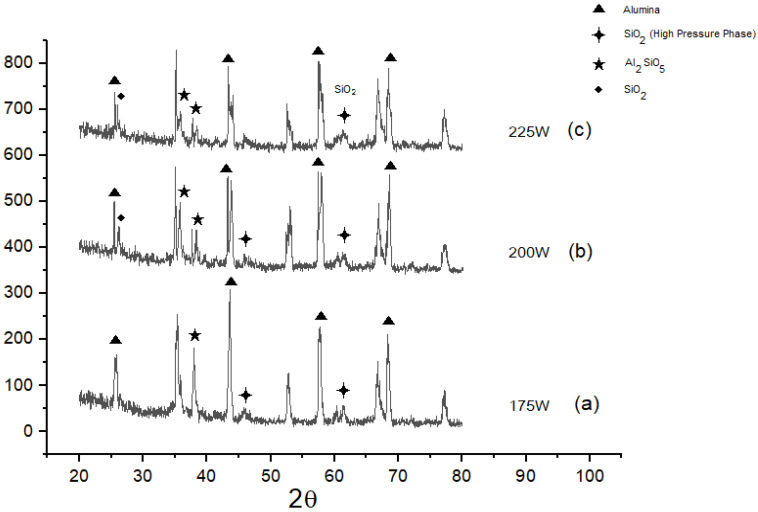
(**a**) XRD for Al_2_O_3_ wt% 90, silicon carbide wt% 10 at 175 W laser power. (**b**) XRD for Al_2_O_3_ wt% 90, silicon carbide wt% 10 at 200 W laser power. (**c**) XRD for Al_2_O_3_ wt% 90, silicon carbide wt% 10 at 225 W laser power.

Laser irradiation is very rapid, and it may also involve the Si sublimation similar to that of high-temperature annealing of SiC, which may leave a carbon-rich surface where mobile C atoms are in abundance [89]. However, as the experiments are conducted in open air, the increase in chemical interaction between SiC and Al_2_O_3_ by varying the laser power or energy density can be analysed in XRD profile changes in Figure 6a–c. The experiments are conducted in open air, which may lead to chemical oxidation of SiO mentioned in Equation (1) into SiO_2_. Similarly, Al_2_SiO_5_ phase in XRD could be due to the formation of an intermediate phase of SiO_2_ and Al_2_O_3_, which is also decreasing with the increase in laser power.

**Figure 7:**
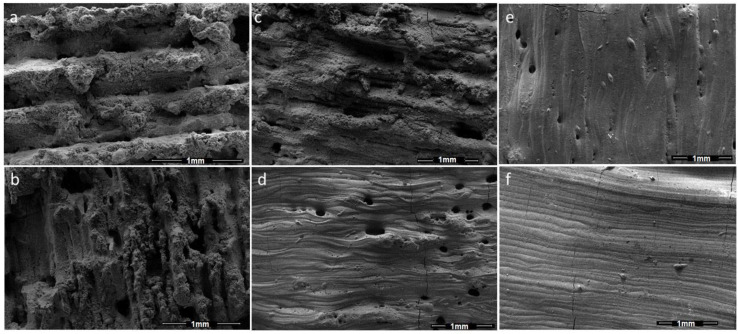
(**a**) Al_2_O_3_ wt% 95, SiC wt% 5 at 120 W laser power. (**b**) Al_2_O_3_ wt% 95, SiC wt% 5 at 150 W. (**c**) Al_2_O_3_ wt% 95, SiC wt% 5 at 180 W. (**d**) Al_2_O_3_ wt% 98, SiC wt% 2 at 120 W. (**e**) Al_2_O_3_ wt% 98, SiC wt% 2 at 150 W. (**f**) Al_2_O_3_ wt% 98, SiC wt% 2 at 180 W.

**Figure 8:**
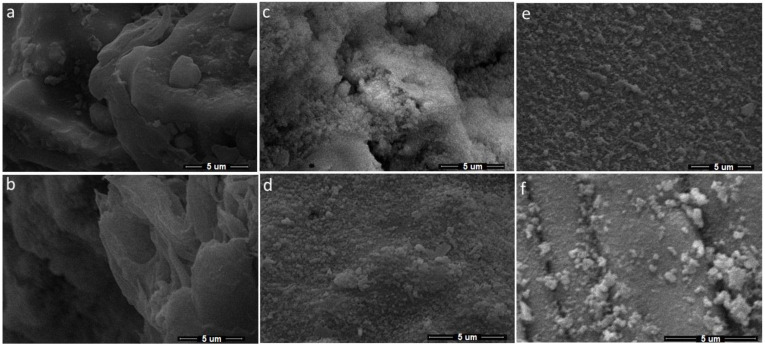
(**a**) Al_2_O_3_ wt% 95, SiC wt% 5 at 120 W laser power. (**b**) Al_2_O_3_ wt% 95, SiC wt% 5 at 180 W. (**c**) Al_2_O_3_ wt% 95, SiC wt% 5 at 150 W. (**d**) Al_2_O_3_ wt% 98, SiC wt% 2 at 120 W. (**e**) Al_2_O_3_ wt% 98, SiC wt% 2 at 150 W. (**f**) Al_2_O_3_ wt% 98, SiC wt% 2 at 180 W.

One phenomenon in PBSLP of Al_2_O_3_ and SiC is the chemical interaction during the process. The other phenomenon is when there is no chemical interaction between the SiC and Al_2_O_3_, as the amount of SiC is decreased. In this case, when SiC is mixed in the specified quantity, it may change the melting temperature of the Al_2_O_3_-SiC mixture, which can be slightly higher than melting temperature of Al_2_O_3_ and slightly lower than the SiC depending on the proportion of the quantity. When the SiC content is increasing, it shows that the melting temperature is also increasing. In Figure 7, two different material proportions (Al_2_O_3_ wt% 95, SiC wt% 5 and Al_2_O_3_ wt% 98, SiC wt% 2, respectively) are presented (each material composition at three different laser powers, 120 W, 150 W and 180 W). The grain in Figure 7f shows partial melting when the SiC content is relatively lower. However, the grains in Figure 7c show sintering with the same laser power. Similarly, the increase in laser power also has the obvious effect of increasing the temperature of the melt pool, which defines the state of PBSLP process from solid-state sintering to partial and complete melting. The solid-state sintering to partial melting can be seen in Figure 7d–f in the samples with the same composition. All the micrographs of Figure 7 can be seen in a more magnified form in Figure 8.

When the laser scan line during PBSLP encounters SiC in bulk or more than usual (as compared to the rest of the material mixture powder layer), it can leave that part un-melted (as the mixing is random), leading to pores clearly showing un-melted morphology on the surface, which can be seen in Figure 8d,e. Even though the melting temperature was higher due to the relatively large amount of SiC in Figure 8a–c, this phenomenon was more abundant within the sintered articles.

**Figure 9:**
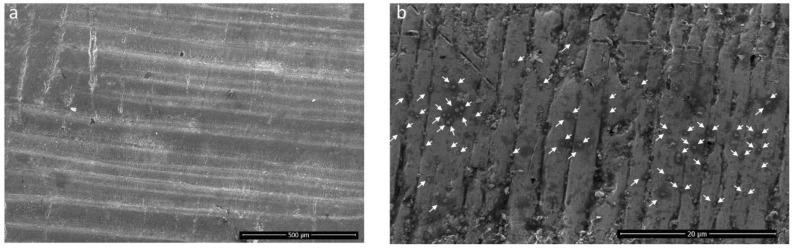
(**a**) Improved surface morphology with crack pinning effect of Al_2_O_3_ wt% 99.5, SiC wt% 0.5 at 120 W laser power. (**b**) Pinning effect of SiC can be seen clearly in magnified micrograph of the same sample in (**a**).

**Figure 10:**
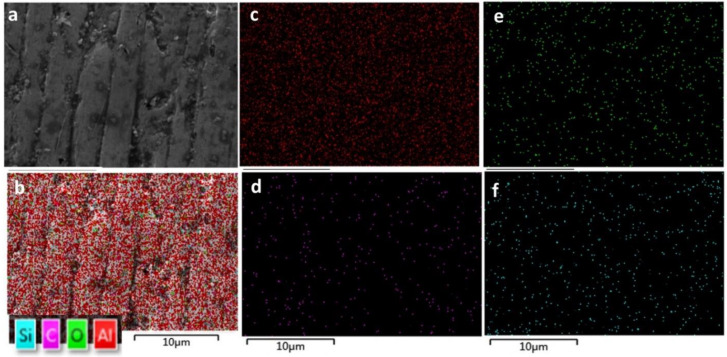
(**a**) Alumina wt% 99.5, silicon carbide wt% 0.5 at 160 W laser power. (**b**) EDS map of Si, C, O and Al; (**c**) EDS map of Al; (**d**) EDS map of C; (**e**) EDS map of C; and (**f**) EDS map of Si of the same article.

Interestingly, when the SiC is in lesser quantities, the un-melted particles of SiC can form a dispersion in the matrix composites. Materials have a clear effect on the crack propagation of the composite, and SiC at the microscopic level can hinder the cracks. Due to the addition of SiC, the un-melted particle (because of the higher temperature of SiC) may exhibit the pinning effect and deflection in crack propagation when used in minimal quantity (Al_2_O_3_ wt% 99.5, SiC wt% 0.5). The crack pinning effects can be seen more clearly in the polished and etched article in Figure 9. Subjected to microscopic examination, the pinning effect can hinder the crack growth trend and crack deflection, or crack bending can shorten the crack in the length of the expansion direction. In more magnified SEM of the same article (Figure 9b), the SiC particles can be visualised in darker regions. An EDS map of the surface of the same article with SiC wt% 0.5 and Al_2_O_3_ wt% 99.5 is also shown in Figure 10.

The crack sensitivity is mainly affected due to the SiC particles in the ceramic mixture, the different elastic modulus E of the heterogeneous matrix and the difference in thermal expansion coefficients. Pinning and deflection occur due to lingering stress field around the two phases and the stress at the crack tip. The melting point, hardness, elastic modulus and thermal expansion coefficient are apparently different for each constituent.

The grain growth inhibition is favourable to the refinement of matrix grains, which eventually promote the densification with improved microstructure and toughening matrix by hindering crack growth. When the SiC particles are in the course of a crack, then crack lines cannot continue to grow. This effect is called crack pinning. Deflection occurs if the crack expansion is large enough to pass through the SiC particles, or if the crack continues to grow around the SiC particle.

**Figure 11:**
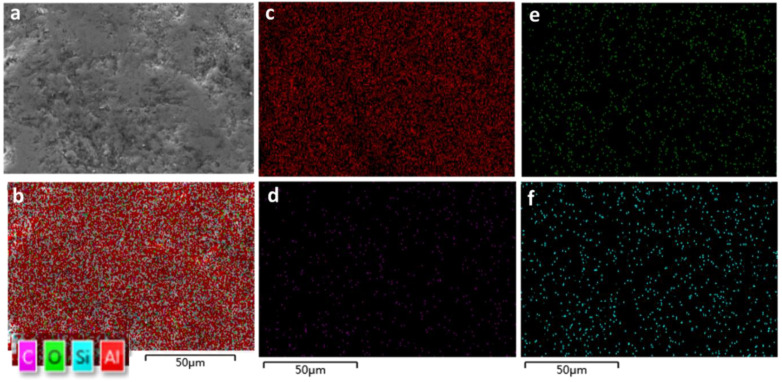
(**a**) Alumina wt% 90, silicon carbide wt% 10. (**b**) EDS map of Si, C, O and Al; (**c**) EDS map of Al; (**d**) EDS map of C; (**e**) EDS map of C; and (**f**) EDS map of Si of the same article.

**Figure 12:**
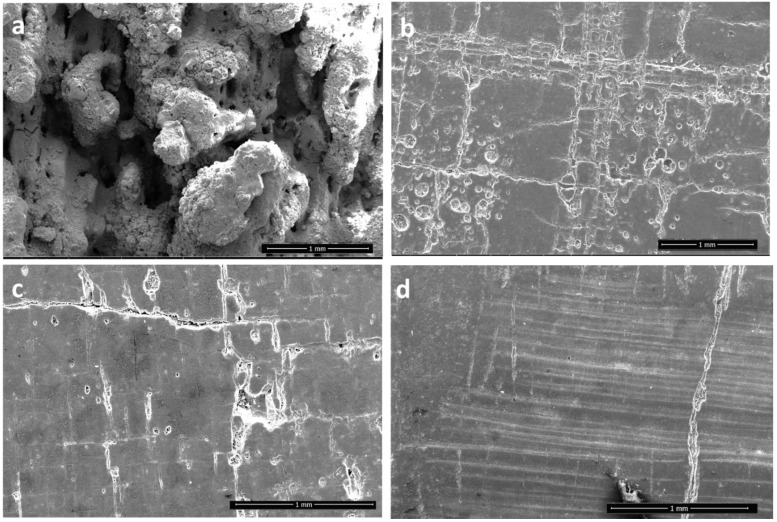
Surface morphology of (**a**) Alumina wt% 80, silicon carbide wt% 20 (unpolished); (**b**) alumina wt% 90, silicon carbide wt% 10 (polished); (**c**) alumina wt% 98, silicon carbide wt% 2 (polished); (**d**) alumina wt% 99.5, silicon carbide wt% 0.5 at 200 W laser power (polished).

An EDS map of the surface of the same article with silicon carbide at wt% 10 and Al_2_O_3_ wt% 90 is shown in Figure 11. The surface morphology of the printed specimen with different loads of silicon carbide are shown in Figure 12.

The printed specimen with alumina wt% 80 and silicon carbide wt% 20 (unpolished) can be seen in Figure 12a, alumina wt% 90 with silicon carbide wt% 10 (polished) in Figure 12b and alumina wt% 98 with silicon carbide wt% 2 in Figure 12c. Lastly, alumina wt% 99.5, silicon carbide wt% 0.5 printed specimen can be seen in Figure 12d. It can be clearly seen that with the reduction in silicon carbide content, the surface morphology keeps improving. When the silicon carbide content is 20 percent, the specimen is prone to cracking and cannot be polished. If subjected to polishing, the sample cracks down completely. The samples with lesser amounts of silicon carbide did not show this behaviour. The porosity and internal defects can also be visualised to be decreasing with the decrease in silicon carbide content. The specimen with the silicon carbide wt% 0.5 has the lowest number of defects.

## 4. Conclusions

In this study, we analysed AM of Al_2_O_3_ and SiC with PBSLP to reduce the cracks by the pinning effect. Furthermore, we carried out an investigation to explore the factors responsible for structural deformation.

At the microscopic level, when the SiC was 2% or less in quantity, it prevented cracks fabricated by PBSLP. The crack pinning effect was more evident when the SiC was in a very small quantity, 0.5%, and evenly distributed.

When the SiC constituent was 10% or higher, it triggered the chemical reaction between Al_2_O_3_ and SiC, which led to structural and surface deformation with a chemical reaction, which may have also resulted in high porosity and unwanted complexion in the manufactured article. The chemical interaction may have also depended on the laser scan steady state. Near the edges of the island, the melt pool was unsteady, showing very low chemical reaction, but when the melt pool became steady, the chemical interaction increased. Both chemical reaction and melt pool stability were interdependent. It is also evident that the hindrance of the melt pool was due to the chemical interaction between the SiC and Al_2_O_3_.

The laser power during the PBSLP of Al_2_O_3_ and SiC led to the obvious effect of solid-state sintering to partial melting and complete melting. The improved properties of Al_2_O_3_ and SiC composite were achieved by adopting the optimised process parameters as per the given weight percentage of the SiC in the composite.

Using very small amounts of SiC reduced cracks during PBSLP of Al_2_O_3_. Future work may focus on the control of the large-scale shrinkage of the article and the cracks at the macroscopic level. During the PBSLP of Al_2_O_3_ and SiC, a composite with a higher amount of SiC (10% or more) may also be possible, but melt pool state control would be required. Future research may focus on the stability of melt pool.

## Figures and Tables

**Table 1:** Chemical component of Al_2_O_3_ powder (wt%) as described by the supplier.

Al_2_O_3_ (wt%)	Na_2_O (wt%)	Fe_2_O_3_ (wt%)	SiO_2_ (wt%)	MgO (wt%)	TiO_2_ (wt%)	CaO (wt%)
99.00	0.0776	0.0124	0.0238	0.0521	0.0035	0.0136

## Data Availability

Not applicable.
